# Aberrant expression of lncRNAs and mRNAs in patients with intracranial aneurysm

**DOI:** 10.18632/oncotarget.13908

**Published:** 2016-12-11

**Authors:** Wen Wang, Hao Li, Lanbing Yu, Zheng Zhao, Haoyuan Wang, Dong Zhang, Yan Zhang, Qing Lan, Jiangfei Wang, Jizong Zhao

**Affiliations:** ^1^ Department of Neurosurgery, Beijing Tiantan Hospital, Capital Medical University, Beijing, China; ^2^ Department of Neurosurgery, The Second Affiliated Hospital of Soochow University, Suzhou, China; ^3^ Beijing Neurosurgical Institute, Capital Medical University, Beijing, China; ^4^ China National Clinical Research Center for Neurological Diseases, Beijing, China; ^5^ Beijing Key Laboratory of Translational Medicine for Cerebrovascular Diseases, Beijing, China; ^6^ Department of Neurosurgery, Zhujiang Hospital, Southern Medical University, Guangzhou, China

**Keywords:** intracranial aneurysm, long non-coding RNA, microarray, gene ontology, pathway analysis

## Abstract

Intracranial aneurysm (IA) is pathological dilatations of the cerebral artery and rupture of IAs can cause subarachnoid hemorrhage, which has a high ratio of fatality and morbidity. However, the pathogenesis of IAs remains unknown. We performed long noncoding RNA (lncRNA) and messenger RNA (mRNA) expression profiles in IA tissues and superficial temporal arteries (STAs). A total of 4129 differentially expressed lncRNAs and 2926 differentially expressed mRNAs were obtained from the microarrays (P < 0.05). Gene ontology (GO) and Kyoto Encyclopedia of Genes and Genomes (KEGG) pathway analyses showed that up-regulated mRNAs were enriched in immune response, inflammatory response, regulation of immune response and lysosome, et al; while the down-regulated mRNAs were enriched in muscle contraction, smooth muscle contraction, cGMP-PKG signaling pathway and vascular smooth muscle contraction, et al. The lncRNA-mRNA co-expression networks were represented in immune response, inflammatory response, muscle contraction and vascular smooth muscle contraction. These findings may gain insight in the pathogenesis of IAs and provide clues to find key roles for IA patients.

## INTRODUCTION

Intracranial aneurysm (IA) is pathological dilatations of the cerebral artery and its overall prevalence is about 3.2% with a mean age of 50 years [1, 2]. Rupture of IAs can cause subarachnoid hemorrhage, which has a high ratio of fatality and morbidity [3]. Although several genome-wide association studies have been performed worldwide [4–7], the pathogenesis of IAs remains unknown.

Long noncoding RNA (LncRNA) is defined as longer than 200 nucleotides and lack of protein-coding ability [8]. Many studies have revealed a wide range of functional activities of lncRNAs and it suggests that some lncRNAs are involved in common cardiovascular diseases, including atherosclerosis, myocardial infarction, and aneurysms, et al [9–11]. ANRIL is identified as a genetic susceptibility locus associated with IA and abdominal aortic aneurysm [12–14]. HIF1a-AS1 is overexpressed in the thoracoabdominal aortic aneurysm and plays a key role in the proliferation and apoptosis of vascular smooth muscle cells in vitro [15]. Therefore, lncRNAs may paly important role in the formation of IA.

In our study, we performed microarrays to investigate lncRNA and messenger RNA (mRNA) expression profiles in aneurismal tissues from IA patients and controls-superficial temporal arteries (STAs). The microarray analysis of the differential lncRNAs and mRNAs is necessary to gain insight in the pathogenesis of the formation and development of IAs.

## RESULTS

### The clinical characteristics of included IA patients

We included 27 IA patients (12 ruptured IAs and 15 unruptured IAs) in our study. The clinical characteristics, including gender, age, IA size, hypertension, smokers and drinkers, were shown in Table 1. There were more patients with hypertension and smokers in ruptured IAs group than those in unruptured IAs group (11 vs. 6; 3 vs. 1). LncRNA and mRNA expression profiles were obtained from 27 samples.

**Table 1:** Clinical characteristics of included patients

Items	Ruptured IAs (n=12)	Unruptured IAs (n=15)	STAs (n=27)
Gender (Female %)	7 (58.33%)	13 (86.67%)	20 (74.07%)
Age (Years, mean±SD)	52.83±6.44	49.87±13.28	51.19±10.89
IA Size (mm, mean±SD)	10.27±7.32	16.97±7.11	ND
Hypertension %	11 (91.67%)	6 (40%)	17 (62.96%)
Smokers %	3 (25%)	1 (6.67%)	4 (14.81%)
Drinkers %	1 (8.33%)	1 (6.67%)	2 (7.41%)

IA, intracranial aneurysm; STA, superficial temporal artery. ND, no data.

### Identification of differentially expressed lncRNAs and mRNAs

**Figure 1:**
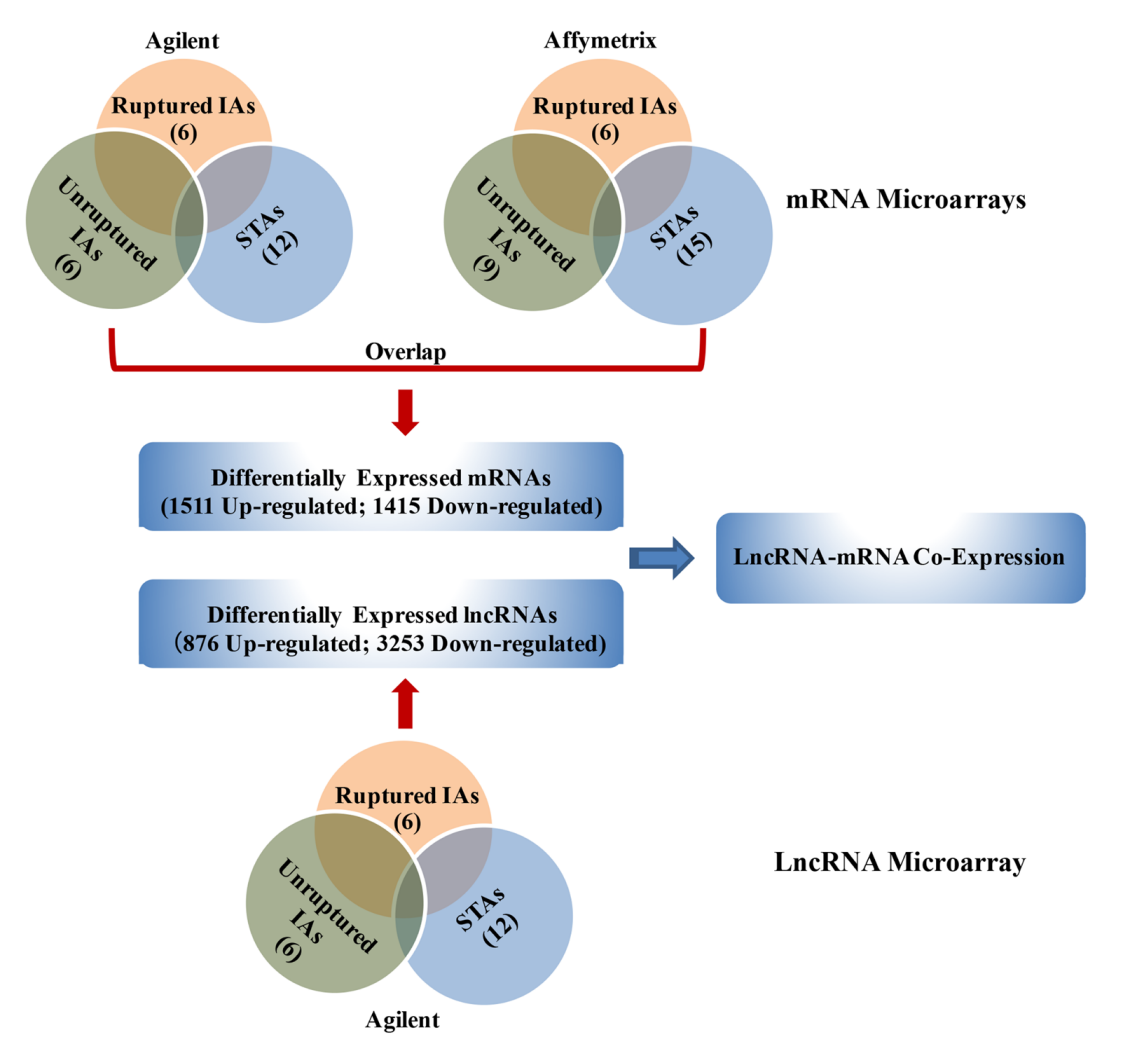
Schematic overview of the workflow IA, intracranial aneurysm; STA, superficial temporal artery.

The workflow of the entire experiment was summarized in Figure 1. We identified 4129 differentially expressed lncRNAs (876 up-regulated; 3253 down-regulated) from the Agilent lncRNA microarrays of 12 IA patients and controls arteries (P < 0.05). Volcano plots were performed to identify differences of lncRNAs (Figure S1A, S1B). A total of 2926 differentially expressed mRNAs were identified from two mRNA microarrays (Agilent, Affymetrix; P < 0.05; Figure S1C-S1F). Of those, 1511 up-regulated mRNAs and 1415 down-regulated mRNAs were screened out in 27 IAs compared with STAs, respectively (Table S1).

**Figure 2:**
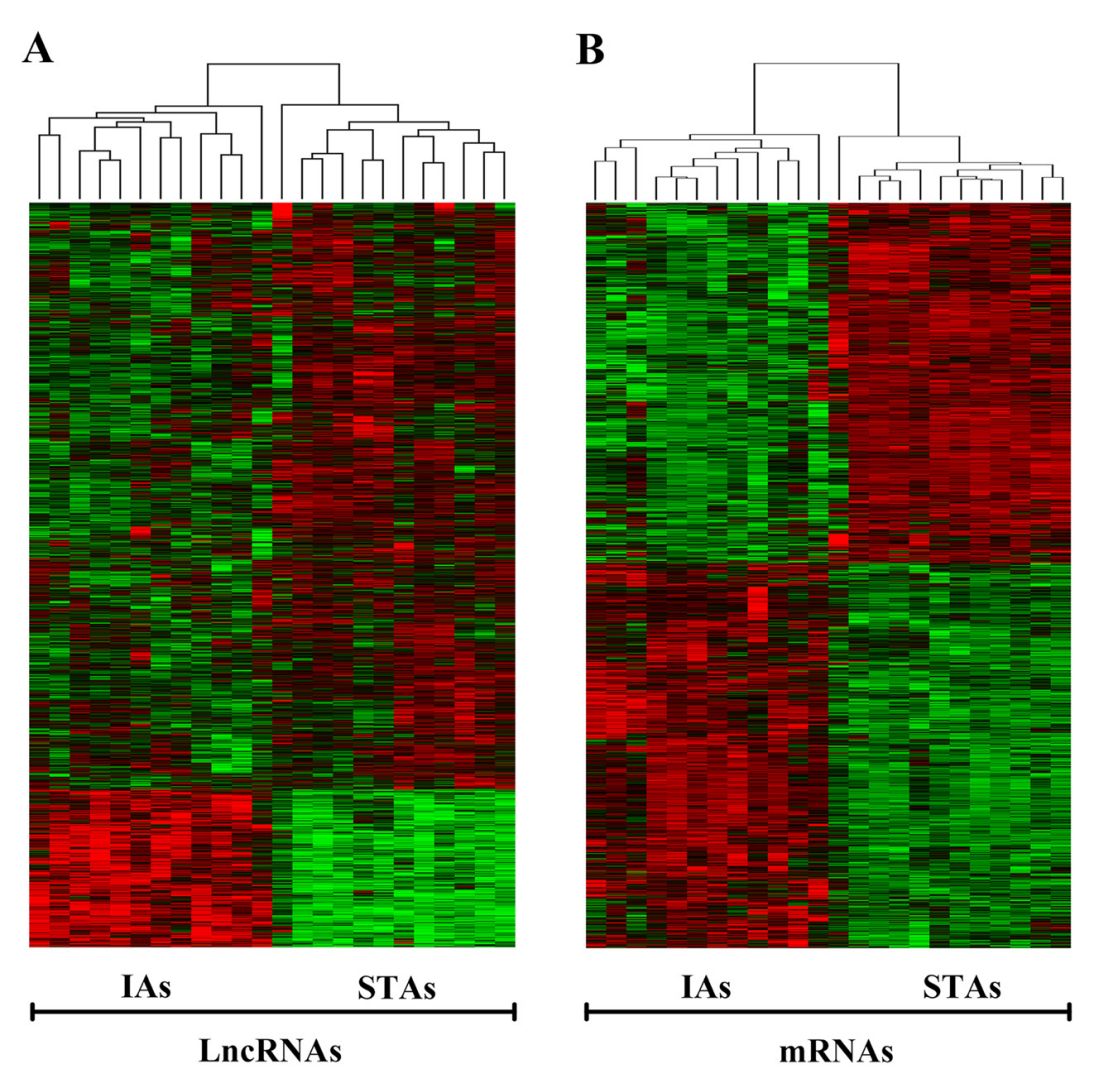
Heat map of differentially expressed lncRNAs and mRNAs of IAs and STAs IA, intracranial aneurysm; STA, superficial temporal artery.

The heat map of the differentially expressed lncRNAs and mRNAs separated by IAs and STAs was showed in Figure 2.

### Function exploration of the differentially expressed mRNAs

**Figure 3:**
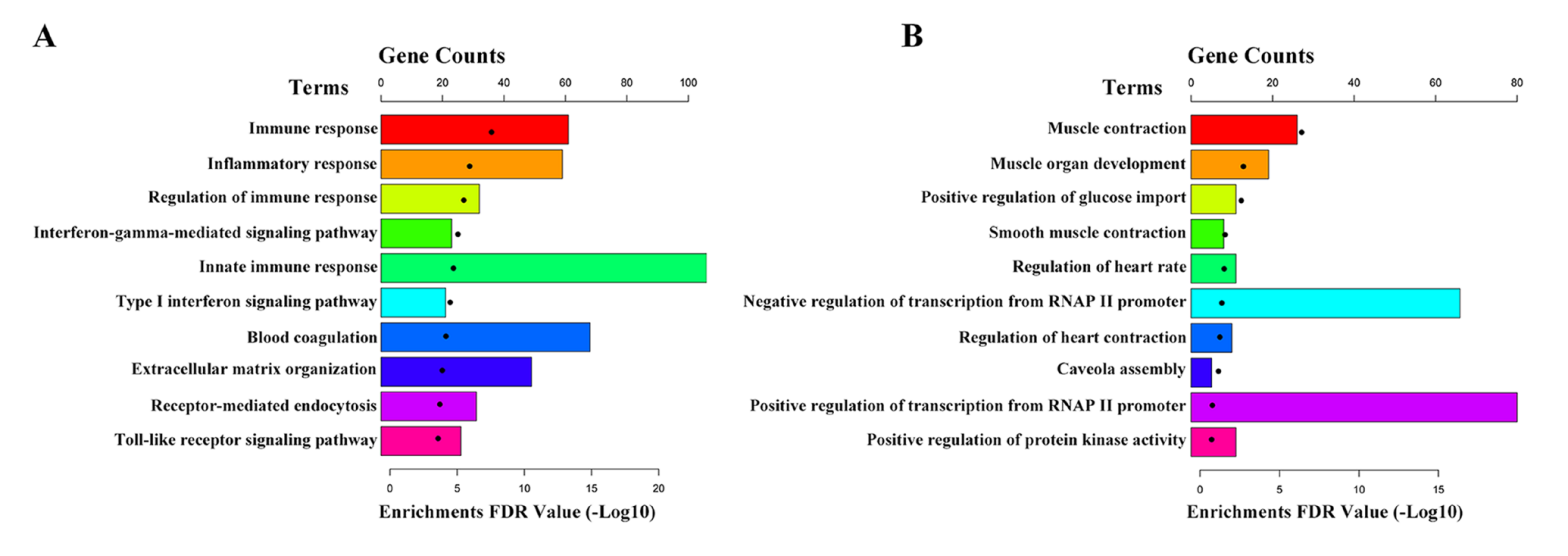
Gene ontology of differentially expressed mRNAs **A.** The top 10 GO terms up-regulated in IAs compared with STAs. **B.** The top 10 GO terms down-regulated in IAs compared with STAs.

**Figure 4:**
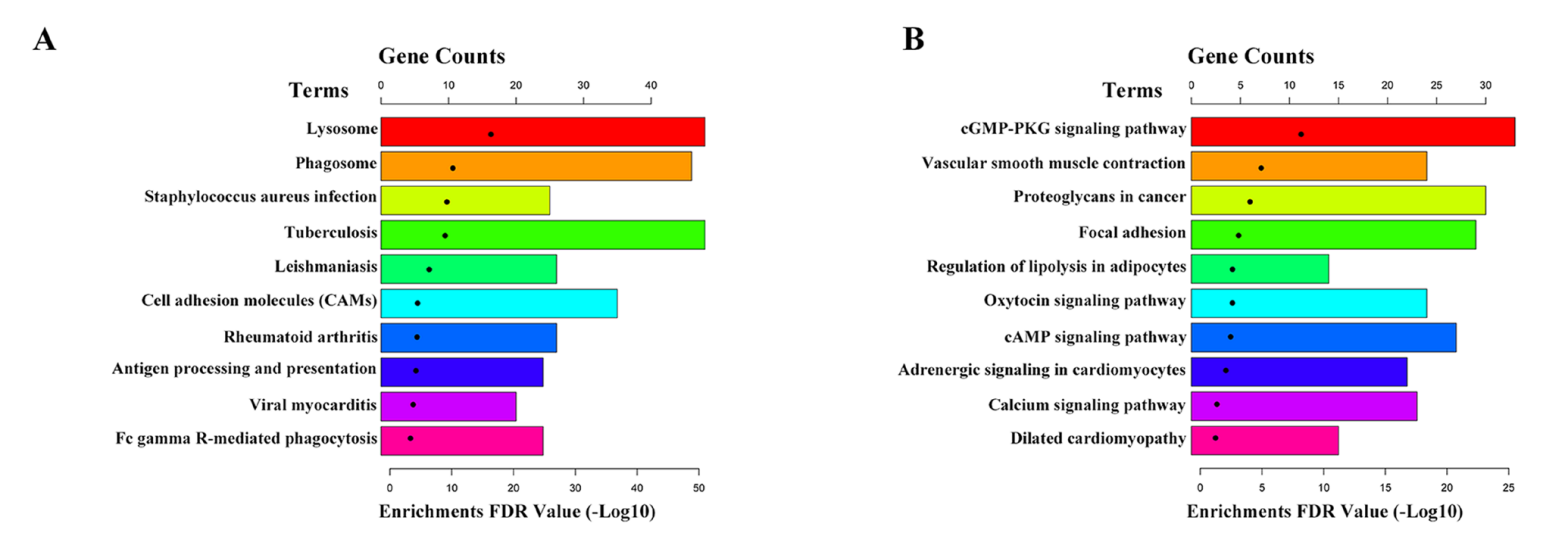
KEGG pathway analysis of differentially expressed mRNAs **A.** The top 10 pathways up-regulated in IAs compared with STAs. **B.** The top 10 pathways down-regulated in IAs compared with STAs.

The 2926 differentially expressed mRNAs were conducted GO and KEGG pathway analyses using DAVID (The Database for Annotation, Visualization and Integrated Discovery,https://david-d.ncifcrf.gov/home.jsp). GO analysis showed that up-regulated genes were enriched in immune response, inflammatory response and regulation of immune response, et al (Figure 3A); while the down-regulated genes were enriched in muscle contraction, muscle organ development, positive regulation of glucose import and smooth muscle contraction, et al (Figure 3B). Moreover, KEGG pathway analysis showed that the up-regulated genes enriched in lysosome, phagosome and staphylococcus aureus infection, et al (Figure 4A); while the down-regulated genes were enriched in cGMP-PKG signaling pathway, vascular smooth muscle contraction and proteoglycans in cancer, et al (Figure 4B).

### LncRNA-mRNA co-expression networks

**Figure 5:**
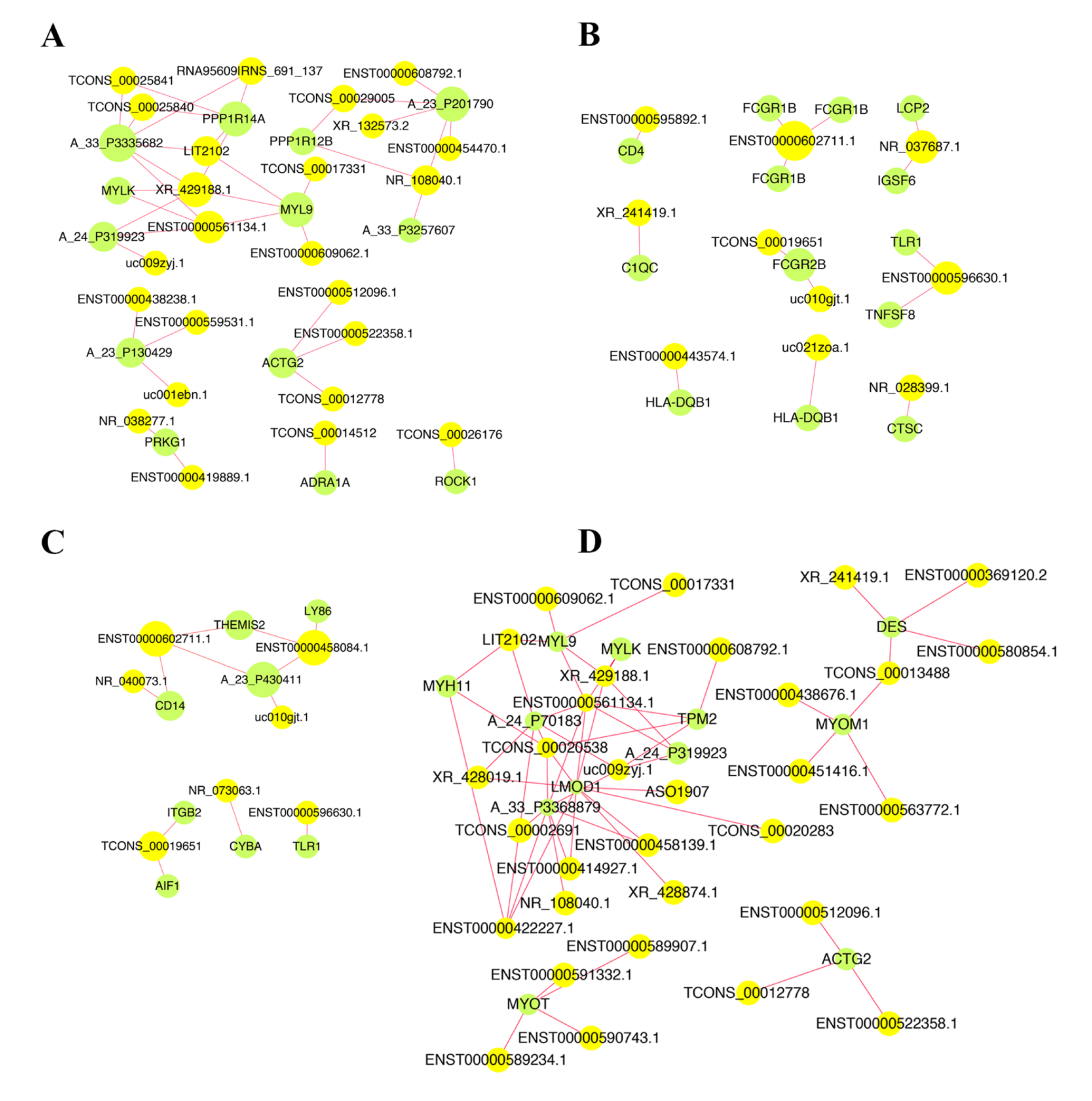
LncRNA-mRNA co-expression networks in four meaningful pathways **A.** 24 lncRNAs interacted with 8 mRNAs in the vascular smooth muscle contraction pathway. **B.** 10 lncRNAs interacted with 10 mRNAs in the GO term of immune response. **C.** 7 lncRNAs interacted with 7 mRNAs in the GO term of inflammatory response. **D.** 31 lncRNAs interacted with 9 mRNAs in the muscle contraction pathway. Yellow, lncRNA; Green, mRNA.

We further performed lncRNA-mRNA co-expression network analysis and the co-expression networks were represented in immune response, inflammatory response, muscle contraction pathway and vascular smooth muscle contraction pathway (Table S2). There were 24 lncRNAs that interacted with 8 mRNAs in the vascular smooth muscle contraction pathway (Figure 5A), 10 lncRNAs interacted with 10 mRNAs in the GO term of immune response (Figure 5B), 7 lncRNAs interacted with 7 mRNAs in the GO term of inflammatory response (Figure 5C) and 31 lncRNAs interacted with 9 mRNAs in the muscle contraction pathway (Figure 5D).

## DISCUSSION

The prevalence of IAs was about 7% in Chinese adults aged 35 to 75 years [16] and the standard treatments are endovascular treatment and open surgery. Many studies have provided evidence of the associations between lncRNA and mRNA [17–19]. However, there are fewer studies focused on the lncRNA-mRNA co-expression study of IAs and the molecular mechanisms behind IA formation remain poorly understood. Therefore, lncRNA-mRNA co-expression analysis could provide clues to detect differentially expressed lncRNAs and mRNAs in meaningful pathways. A total of 575 lncRNAs and 110 mRNAs were included in networks of four meaningful pathways.

The neighboring mRNAs tend to have high correlation with lncRNAs in IAs. We studied the genomic position of differentially expressed lncRNA and mRNA to identify the nearest coding genes in our microarray dataset. Moreover, we’ve redone the lncRNAs-mRNAs (4129 lncRNAs, 2926 mRNAs) co-expression analysis for IAs. Surprisingly, 47.33% of IA mRNAs were adjacent to lncRNAs within 1KB up- and downstream of lncRNAs (Correlation coefficient (Cor) ≥ 0.9 or ≤ -0.9; P < 0.05). Even focusing on the Cor ≥ 0.8 or ≤ -0.8, there were still about 29.85% of the nearest mRNAs surrounded by lncRNAs. The genomic position and orientation of lncRNAs and mRNAs do have correlation with their Cor value. LncRNAs may regulate the expression of neighboring mRNAs at the level of chromatin modification, transcription and post-transcriptional processing [9, 20]. Some positive/negative correlations of lncRNAs and neighboring mRNAs were observed in the microarray. LncRNAs can regulate transcription by acting as enhancers or co-factors and they can influence gene promoters through interacting with initiation complex [21–23]. Moreover, some lncRNAs, like antisense lncRNAs, have the ability to regulate mRNAs expression through splicing, editing and translation in the post-transcriptional processing [24, 25].

It reported that AGTR1 could regulate blood pressure and promote angiogenesis [26]. MYLK can encode smooth muscle and nonmuscle isoforms. Causal mutation of MYLK was identified in thoracic aortic aneurysm patients [27]. Recently, Yan et al. performed a genetic study of IAs and it indicated that ADAMTS15 might have antiangiogenic activity [4]. Therefore, these genes played important roles in regulating vascular smooth muscle contraction pathway (Figure 5A). Besides, the differentially expressed microRNAs (miRNAs) were crucial to the pathophysiology of IAs [6, 28]; the miRNA/mRNA profiling and regulatory network of IAs also showed similar results in vitro or in vivo [7, 29]. Serum miRNAs might be novel biological markers that were useful in assessing the likelihood of IAs occurrence and development [30]. These miRNAs also have warning effect for the rupture [31].

The vascular remodeling and inflammation response played important roles in the pathogenesis of IAs [32, 33]. The rupture of IAs often lead to extremely high morbidity and mortality [34]. It reported immune response and inflammatory response were more enhanced in ruptured aneurysms [35–37]. Macrophage infiltration and M1/M2 imbalance were strongly associated with aneurysm rupture [38, 39]. Smooth muscle cells (SMCs) migrated into the intima and produced myointimal hyperplasia due to endothelial injury and proliferation [40]. Concurrently, SMCs underwent phenotypic modulation to a proinflammatory phenotype which led to IA formation [41]. Therefore, we overlapped the differentially expressed mRNAs between ruptured IAs or unruptured IAs and STAs to avoid the genetic basis of their development.

There are limitations in our study. Agilent mRNAs profile was performed on 12 IA patients and Affymetrix mRNA profile was performed on 15 patients, so many differentially expressed mRNAs may be filtered due to the different platforms. However, the mRNAs were isolated from both Agilent and Affymetrix platforms to enlarge the number of samples and increase the statistical power. The identified mRNAs of two microarrays overlapped were more likely to be differentially expressed genes. STAs were selected because of the difficulties in obtaining intracranial arteries from humans and they have been widely used as controls in previous studies [42–44]. The morphological and phenotypic differences between STAs and intracranial arteries may affect the final results, which caused inevitable bias. Moreover, this study was mainly based on microarray analysis and bioinformatics analysis. LncRNAs involved in the identified pathways may further narrow the scope of future explorations. Therefore, further studies were needed to confirm these lncRNAs functions with knockdown and over-expression experiments. The manuscript was our preliminary work and future work remains to be done.

In conclusion, we identified 4129 differentially expressed lncRNAs and 2926 differentially expressed mRNAs from the microarrays of IA patients and STAs. The co-expression networks also indicated the correlations between lncRNAs and mRNAs in IA patients. These findings may partly explain the pathogenesis of the formation and development of IAs and provide clues to find key roles for IA patients.

## MATERIALS AND METHODS

### Patients and samples

We enrolled 27 IA patients who were diagnosed with saccular cerebral aneurysms and underwent microsurgical clipping in Beijing Tiantan Hospital. Further, we collected 27 STAs that were injured during the pterional craniotomies and lateral frontal craniotomies in our study. The informed consent was obtained at enrollment and our study was approved by the Ethics Committee in Beijing Tiantan hospital. The study was carried out in accordance with the Declaration of Helsinki and all methods were performed in accordance with the relevant guidelines and regulations.

The clinical characteristics of all IA patients are summarized in Table 1. We obtained aneurismal and STAs tissues in operations and stored at -80°C freezer until RNA extraction. RNA was extracted by using TRIzol reagent (Invitrogen, Grand Island, NY, USA) according to the manufacturer's instructions. The quality evaluation was determined by using Spectrophotometer (NanoDrop ND-1000) and Agilent 2100 Bioanalyzer (Agilent Technologies, Santa Clara, CA, USA).

### LncRNA and mRNA microarrays

The Agilent Human 4 × 180K lncRNA and mRNA Microarrays (Agilent, Santa Clara, CA, USA) were performed on 12 IA tissues and 12 STAs by Gene Expression Hybridization Kit (Agilent, Santa Clara, CA, US) according to the manufacturer's instructions. The Affymetrix Human Genome U133 GeneChip Microarrays (Affymetrix, Santa Clara, California) were performed on 15 IA tissues and 15 STAs according to the manufacturer's instructions. We selected several genes randomly and examined their expression levels with quantitative real-time polymerase chain reaction (qRT-PCR). The qRT-PCR results matched well with the microarray data. The microarray data can be obtained at the Gene Expression Omnibus (GEO) database (GSE75436;http://www.ncbi.nlm.nih.gov/geo). Analyses of the arrays were performed using R software (version 3.2.3).

The differentially expressed lncRNAs and mRNAs were filtered by at least P < 0.05 and false discovery rate (FDR) < 0.05. To avoid the factors of IA rupture, we compared the differentially expressed lncRNAs and mRNAs between ruptured IA and unruptured IA with STAs, respectively (Figure 1).

### Statistical analysis

All statistical data was analyzed by using SPSS (version 22; SPSS Inc., Chicago, IL, USA). A two-sided P value of < 0.05 was regarded as statistically significant. The raw data was normalized using Quantile Algorithm in Gene Spring Software 13.0 (Agilent technologies, CA, USA).

Gene ontology (GO) and Kyoto Encyclopedia of Genes and Genomes (KEGG) pathway analyses were performed by DAVID (The Database for Annotation, Visualization and Integrated Discovery,https://david-d.ncifcrf.gov/home.jsp).

## SUPPLEMENTARY MATERIALS AND TABLES






