# Supplementary material for: Aberrant expression of lncRNAs and mRNAs in patients with intracranial aneurysm
Source: Oncotarget. 2016 Dec 11;8(2):2477–84. doi: 10.18632/oncotarget.13908 (PMC5356817; doi:10.18632/oncotarget.13908)
Supplement: Supplementary file 1 [file oncotarget-08-2477-s001.pdf]

Aberrant expression of lncRNAs and mRNAs in patients with intracranial aneurysm

Supplementary Material

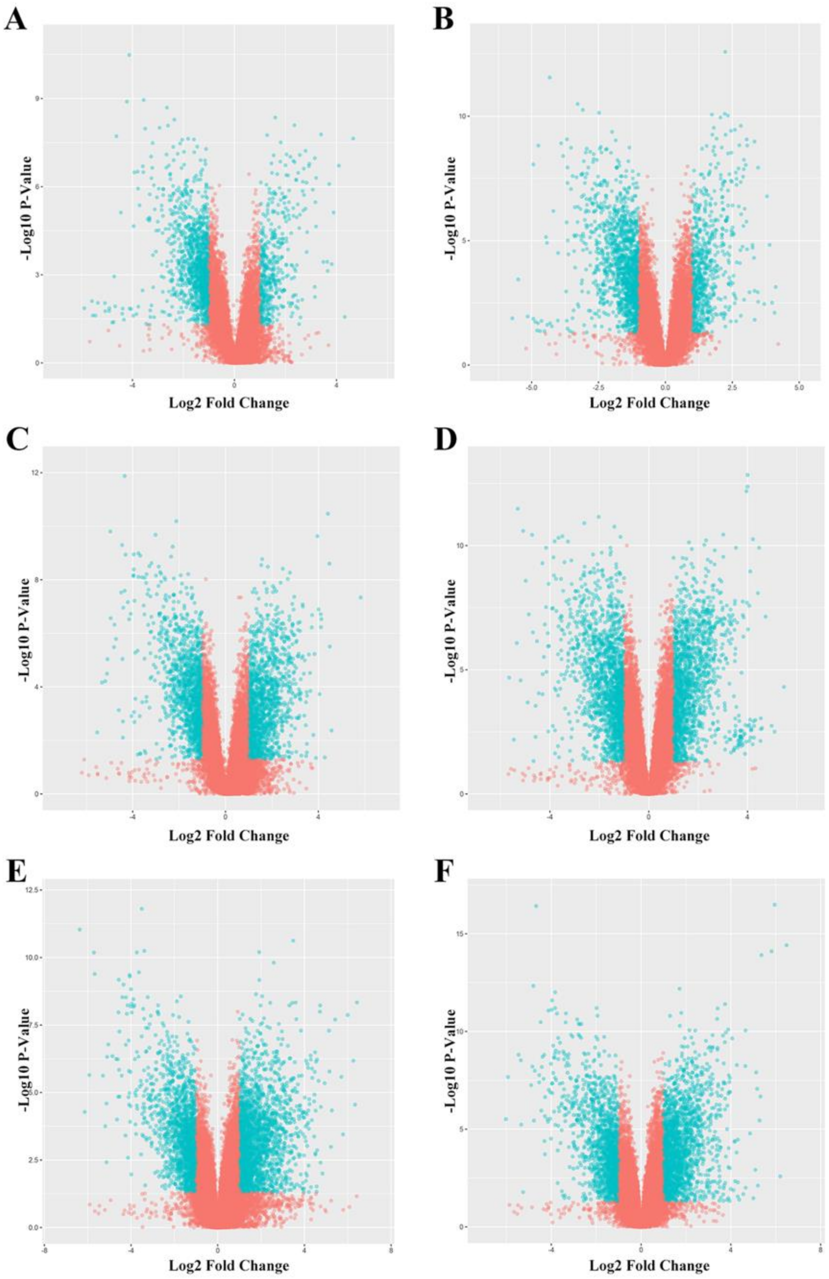

Figure S1. LncRNA and mRNA expression profiles in ruptured or unruptured IAs and STAs. A. Volcano plots of lncRNAs expression between ruptured IAs and STAs in Agilent microarray. B.

Volcano plots of lncRNAs expression between unruptured IAs and STAs in Agilent microarray. C. Volcano plots of mRNAs expression between ruptured IAs and STAs in Agilent microarray. D. Volcano plots of mRNAs expression between unruptured IAs and STAs in Agilent microarray. E. Volcano plots of mRNAs expression between ruptured IAs and STAs in Affymetrix microarray. F. Volcano plots of mRNAs expression between unruptured IAs and STAs in Affymetrix microarray. The green dots represented differentially expressed genes ($P < 0.05$; Fold Change > 2.0). IA, intracranial aneurysm; STA, superficial temporal artery.

For Supplementary Tables 1-2 see Supplementary Files.

Table S1. Identification of differentially expressed lncRNAs and mRNAs.

Table S2. Significant correlation of lncRNAs and mRNAs in the four meaningful pathways.
